# Splenic Anomalies of Shape, Size, and Location: Pictorial Essay

**DOI:** 10.1155/2013/321810

**Published:** 2013-04-21

**Authors:** Adalet Elcin Yildiz, Macit Orhan Ariyurek, Musturay Karcaaltincaba

**Affiliations:** Department of Radiology, Faculty of Medicine, Hacettepe University, 06100 Ankara, Turkey

## Abstract

Spleen can have a wide range of anomalies including its shape, location, number, and size. Although most of these anomalies are congenital, there are also acquired types. Congenital anomalies affecting the shape of spleen are lobulations, notches, and clefts; the fusion and location anomalies of spleen are accessory spleen, splenopancreatic fusion, and wandering spleen; polysplenia can be associated with a syndrome. Splenosis and small spleen are acquired anomalies which are caused by trauma and sickle cell disease, respectively. These anomalies can be detected easily by using different imaging modalities including ultrasonography, computed tomography, magnetic resonance imaging, and also Tc-99m scintigraphy. In this pictorial essay, we review the imaging findings of these anomalies which can cause diagnostic pitfalls and be interpreted as pathologic processes.

## 1. Normal Anatomy of the Spleen

Spleen is an intraperitoneal organ located in the left upper quadrant with a smooth serosal surface. Its normal position is provided by two fatty ligaments: the gastrosplenic ligament, which connects the greater curvature of the stomach to the ventral aspect of the spleen, and the splenorenal ligament between the left kidney and the spleen, attaching the spleen to the posterior abdominal wall. The splenic hilum is directed anteromedially and includes splenic artery and six or more branches of the splenic vein. Splenic size changes according to the age and weight. Configuration of the spleen is also variable according to the indentations of the organs including stomach, colon, pancreas, and kidney which are in close relation to the spleen [1–4].

## 2. Splenic Clefts, Notches, and Lobulations

**Figure 1:**
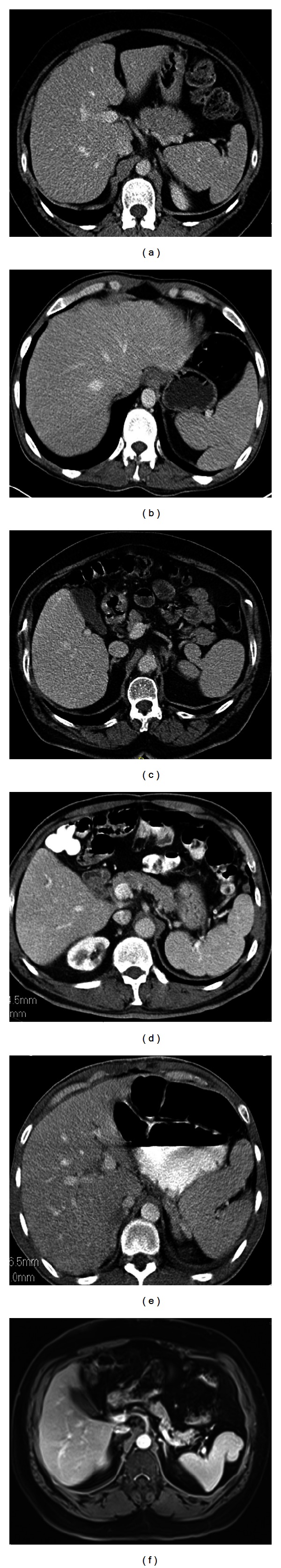
Axial contrast enhanced CT images (a)–(e) and post-contrast T1-weighted MR image (f) show examples of different type of persisting splenic fetal lobules and shape variations, which were incidentally detected in six different patients. Note the close relation of the medially oriented splenic lobules with upper pole of the left kidneys in (a) and (c).

The fetal spleen is lobulated, and these lobules normally disappear before the birth. Lobulation of the spleen may persist into adult life and be typically seen along the medial part of the spleen. A persisting lobule results in a variation in shape of the spleen (Figure 1). But sometimes the lobule may extend medially anterior to the upper pole of the left kidney and less often posterior to the upper pole of the left kidney. Although these lobules are not of any clinical importance for the patient, close relation of the splenic lobule to the upper pole of the left kidney may cause misinterpretations as a mass originating from the kidney by the radiologists [1, 2]. 

**Figure 2:**
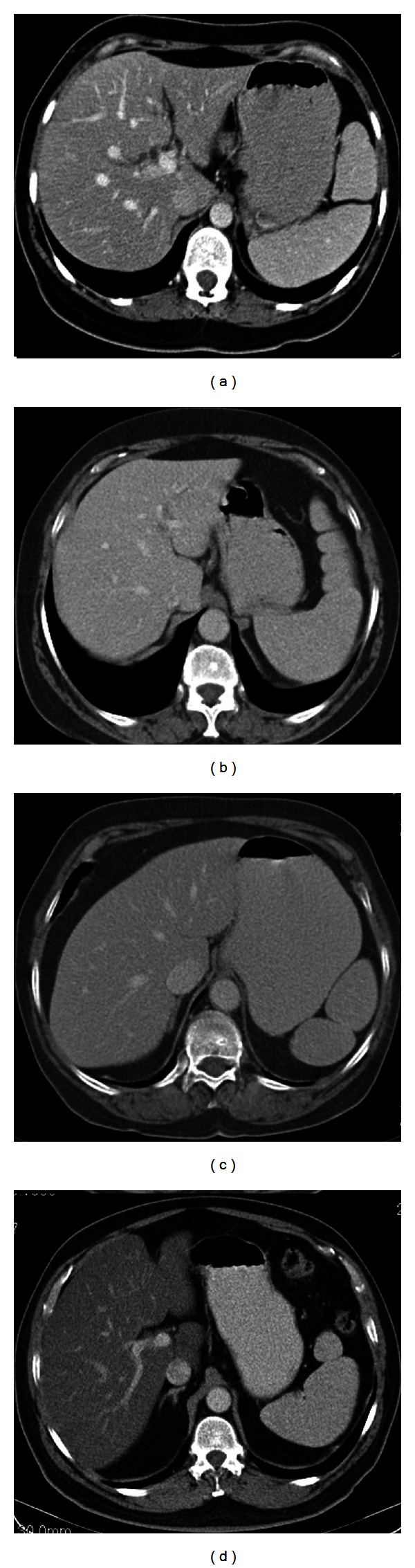
Axial contrast enhanced CT images show examples of splenic clefts in four different patients which were incidentally detected. In (b) there are also coexisting fetal lobules at the medial aspect of the spleen.

The notches and clefts of the spleen are other congenital shape anomalies which are located on the diaphragmatic surface and especially superior border of the adult spleen. These are remnants of the grooves that originally separate the fetal lobules. Clefts occasionally may reach 2-3 cm in length and may cause misinterpretations as a splenic laceration in patients with abdominal trauma [1, 2] (Figure 2). Persistence of this appearance on delayed images of dynamic contrast enhanced CT is useful in diagnosing clefts, whereas in case of trauma these clefts fill with contrast.

## 3. Fusion and Location Anomalies

**Figure 3:**
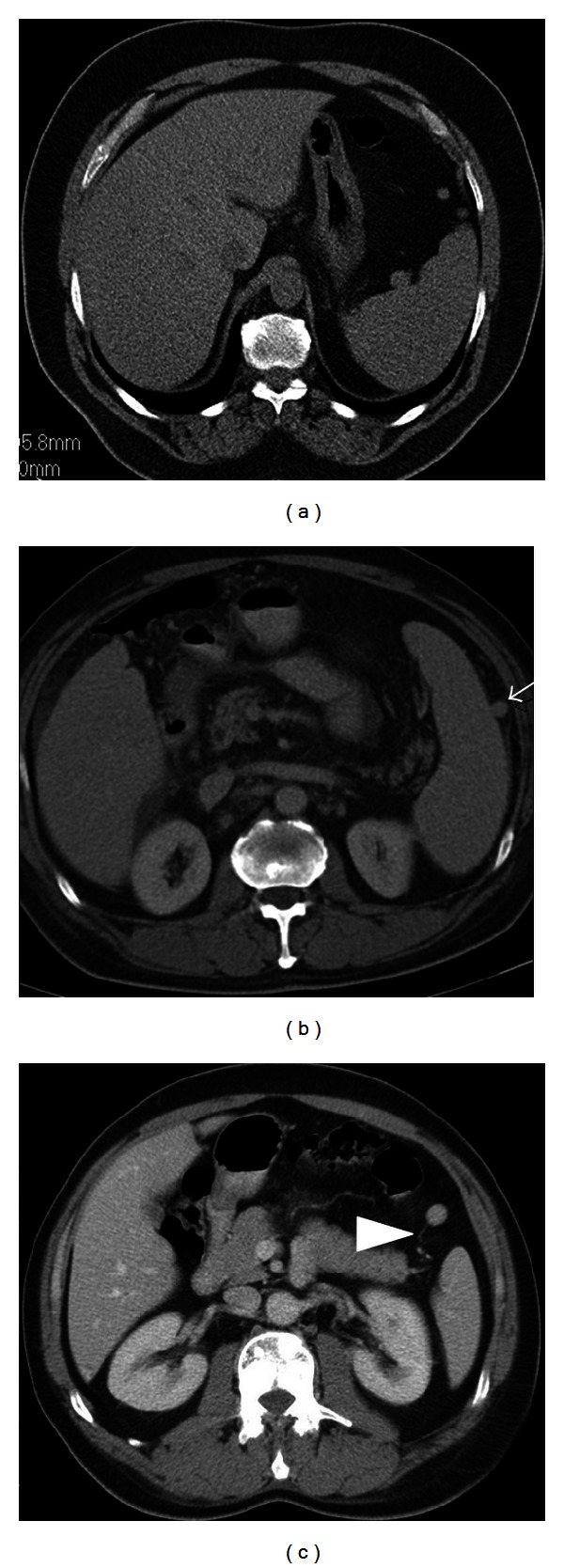
Axial nonenhanced (a) and contrast enhanced (b, c) images show accessory spleens in three different patients. In (a), a 51-year-old male patient has three accessory spleens which are located at splenic hilum and anterior to the spleen. In (b), a 59-year-old male patient has an accessory spleen located at the lateral aspect of the spleen (arrow). In (c), a 50-year-old male patient has an anteriorly located accessory spleen; note its vascular supply originating from splenic artery (arrowhead).

**Figure 4:**
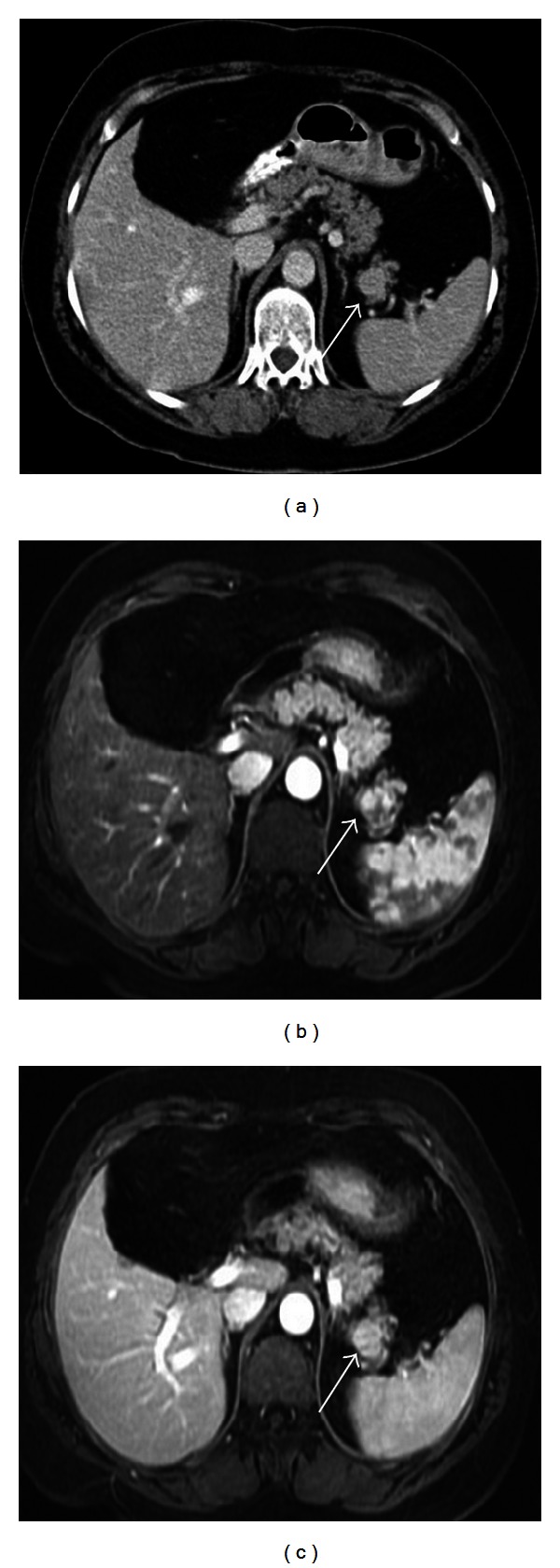
Axial contrast enhanced CT image (a), contrast enhanced T1-weighted image at arterial (b) and venous phases (c) show an intrapancreatic nodular mass in a 63-year-old female patient. The mass has similar density at CT and contrast enhancement pattern at MR images with the spleen indicating an intrapancreatic accessory spleen.

**Figure 5:**
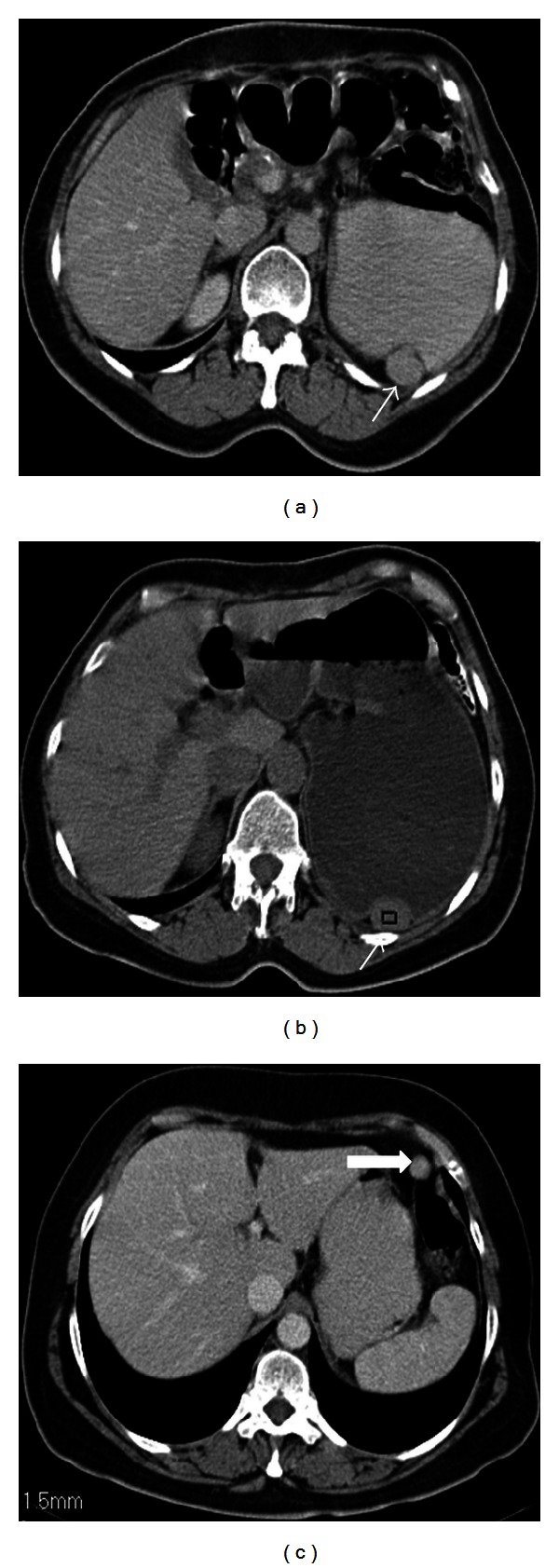
Accessory spleens can locate at the wall of stomach or bowel. Axial contrast-enhanced CT image (a) with oral contrast medium shows a nodular hyperdense lesion (arrow) indenting the posterior wall of the stomach in a 57-year-old female patient. The follow-up axial nonenhanced image (b) with oral water shows persistence of its high density indicating an accessory spleen. Axial contrast enhanced CT image (c) shows an accessory spleen located closely to the colonic wall (thick arrow).

**Figure 6:**
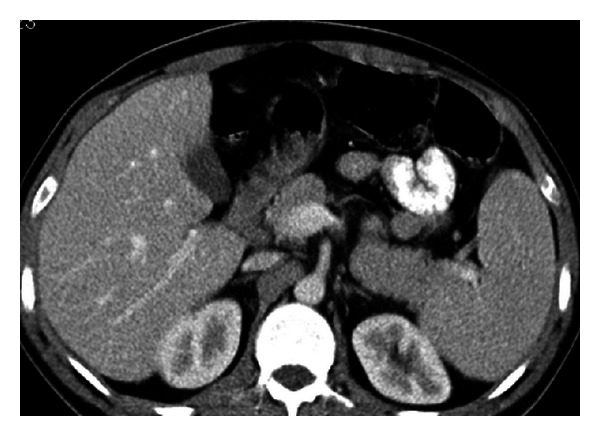
Axial contrast enhanced CT image of a 29-year-old male patient shows splenopancreatic fusion anomaly.

Accessory spleen, in other words supernumerary spleens, splenunculi, or splenules, results from the failure of fusion of the primordial splenic buds in the dorsal mesogastrium during the fifth week of fetal life. Incidence of accessory spleen in the population is 10%–30% of patients in autopsy series and 16% of patients undergoing contrast enhanced abdominal CT. Although the most common location for an accessory spleen is splenic hilum (75%) (Figure 3) and pancreatic tail (25%) (Figure 4), it can occur anywhere in the abdomen including gastrosplenic or splenorenal ligaments, wall of stomach or bowel (Figure 5), greater omentum or the mesentery, and even in the pelvis and scrotum. Accessory spleen usually measures 1 cm in diameter, but its size varies from a few milimeters to centimeters. Also the number of accessory spleens can vary from one to six [1, 5–7]. Accessory spleens are usually incidentally detected and asymptomatic, but in case of unexpected locations, accessory spleen can be of clinical importance. In malignancy patients, an unexpected location of an accessory spleen can be misinterpreted as a metastatic lymph node. But the identical imaging findings of the accessory spleen with the normal splenic tissue on CT and MRI can be helpful for the differential diagnosis. Also demonstration of feeding artery from splenic artery and use of iron containing contrast agents can be helpful for diagnosis (Figure 3). In patients with splenic trauma, an accessory spleen may become clinically important to preserve splenic tissue in case of splenectomy. But in a patient who had splenectomy for hypersplenism, a preservable splenic tissue is an undesired condition that may cause recurrent disease. Splenogonadal fusion anomaly may mimic tumors and result in unnecessary surgeries. So it is important to characterize this anomaly as noninvasively as possible by using ultrasonography, CT, MRI, and Tc-99 m sulfur colloid scintigraphy. Splenopancreatic fusion anomaly which is a form of splenopancreatic field abnormalities including ectopic splenic tissue in the pancreatic tail and ectopic pancreatic tissue in the spleen or accessory spleen may also be detected incidentally (Figure 6). This rare anomaly may result from disturbances in the embryogenesis; thus, both organs arise from dorsal mesogastrium near each other. During the embryogenesis, the close interaction between pancreatic tail and splenic hilum may result in a fusion. The clinical importance of this rare anomaly is to avoid possible complications when splenectomy or distal pancreatectomy is planned [1, 6, 8].

Wandering or ectopic spleen is a rare entity in which the spleen is located outside of its normal location. Its reported incidence in several large series of splenectomies is less than 0.5% and mainly detected in children and women between 20 and 40 years of age. The reason for the wandering spleen is the laxity or maldevelopment of the supporting splenic ligaments, and the spleen can be found in any part of the abdomen related to the length of the vascular pedicle.

**Figure 7:**
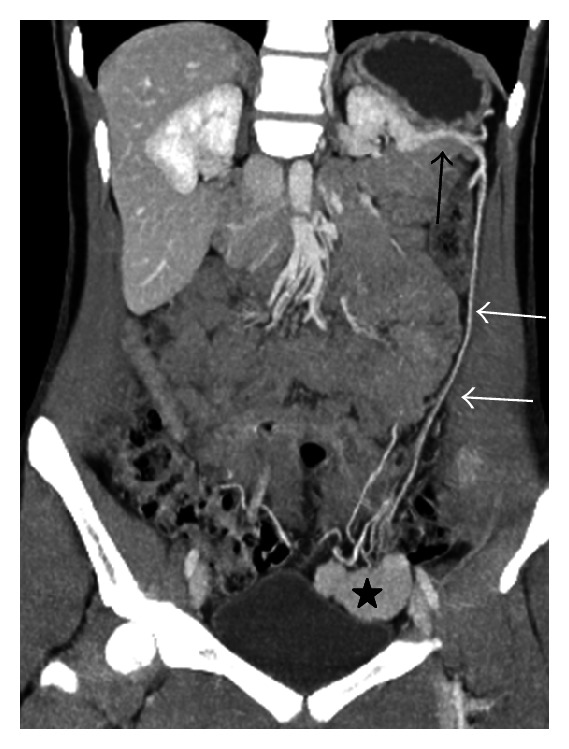
Coronal venous phase MIP image shows a wandering spleen (star) located at pelvis and its vascular pedicle (white arrow) originating from splenic artery (black arrow).

**Figure 8:**
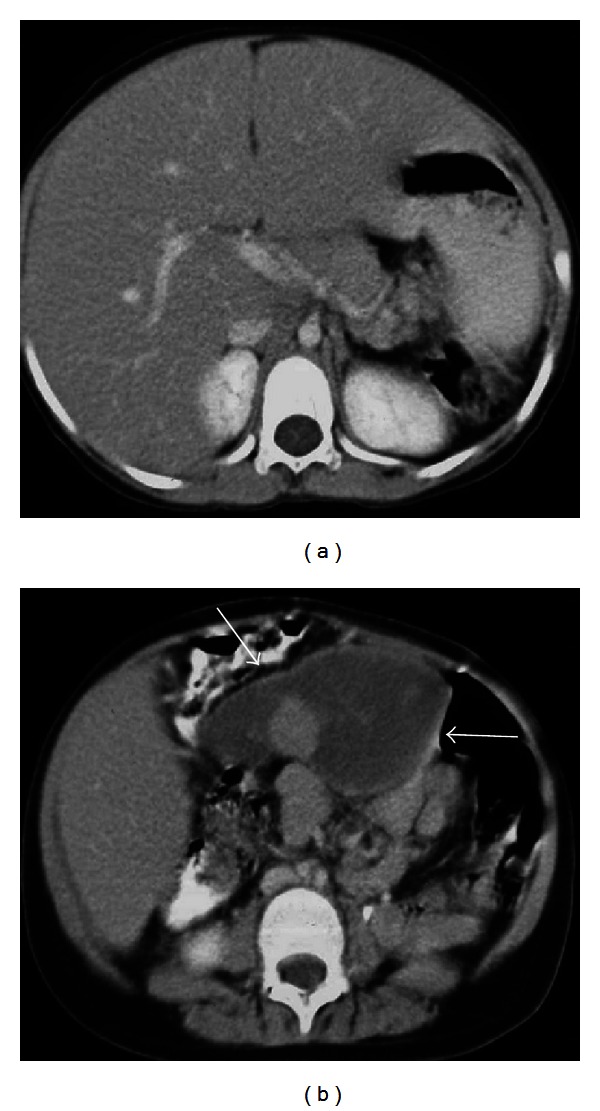
Axial contrast enhanced CT images of a 19-year-old female patient who was presented with acute abdominal pain. There is no splenic tissue at left upper quadrant (a) and a heterogenously hypodense mass (arrows) of a torsioned wandering spleen at midline (b) which was proven surgically. Hypodense parenchyma indicates infarction.

Wandering spleen may be incidentally detected or may cause different degrees of abdominal pain related to acute, chronic, and intermittent torsion of the vascular pedicle. Ultrasonography and CT are the most used methods for diagnosis. Imaging findings of wandering spleen are the absence of the spleen in its normal position and a mass located anywhere in the abdomen or pelvis with enhancement pattern of a normal splenic tissue (Figure 7). In case of torsion, a “whirl” appearance of its twisted pedicle and impaired enhancement of the mass can also be helpful. The treatment choice of a wandering spleen is splenopexy. Splenectomy is required only in case of infarction, which can be diagnosed radiologically. Doppler ultrasonography and contrast enhanced CT can be used to evaluate splenic vascularization. In splenic torsion, doppler ultrasonography shows no flow within the spleen and a low diastolic velocity with an elevated resistive index in the proximal splenic artery. Contrast enhanced CT can show a total absence of or heterogeneous enhancement pattern within the spleen related to partial or total infarction (Figure 8). If there is a contraindication for contrast enhanced CT, findings of infarction on unenhanced CT are low attenuation of the spleen relative to the liver, a hyperdense intraluminal filling defect in the splenic vessels indicating an acute thrombus, and high density of the splenic capsule compared with the parenchyma (“rim” sign) [1, 6, 9, 10]. 

## 4. Polysplenia and Asplenia

Polysplenia and asplenia typically occurs in association with situs ambiguous which is also known as heterotaxia. Situs ambiguous with asplenia (Ivemark syndrome), also referred to as right isomerism or bilateral right sidedness, is characterized by ambiguous location of the abdominal organs and congenital absence of most or all of normal splenic tissues. Greater prevalence of reported cases is in males, and congenital heart disease is present in most or all cases. Most affected individuals often die within the first year of their life because of related severe congenital heart disease and immunodeficiency related to absence of the spleen.

**Figure 9:**
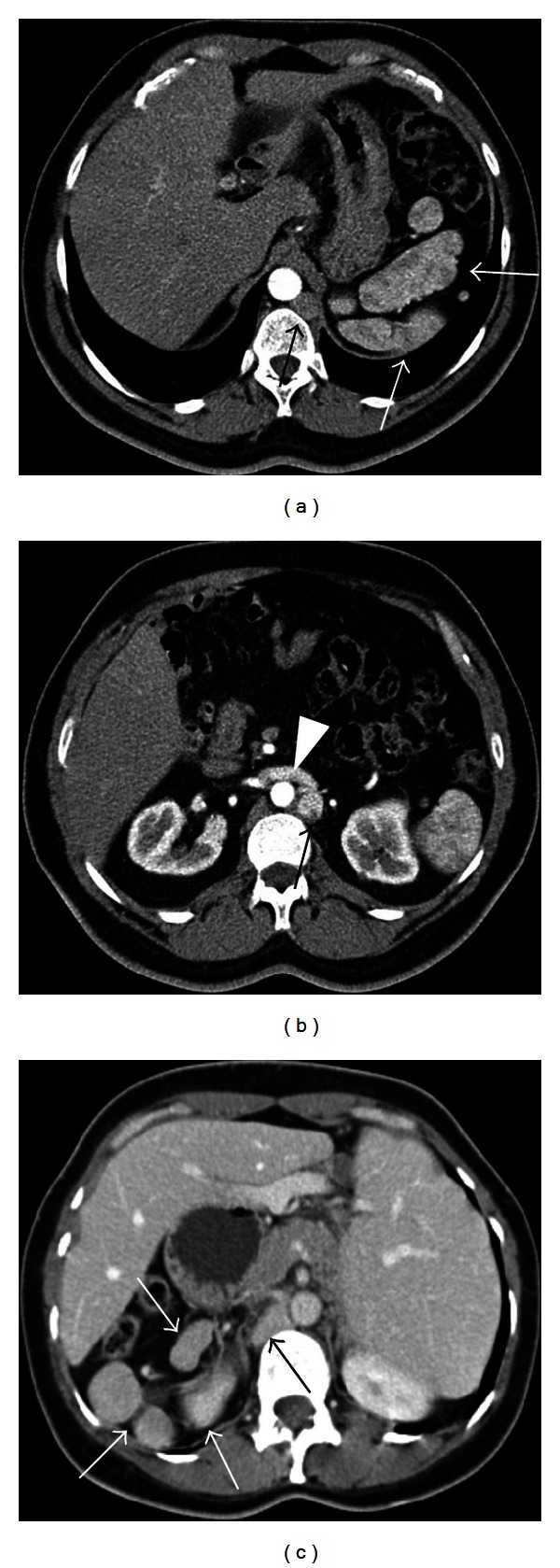
Heterotaxy syndrome in 48-year-old male (a), (b) and 49-year-old female patients (c). Axial contrast enhanced CT images (a), (b) show polysplenia (white arrows) at left upper quadrant which have heterogenous enhancement pattern on arterial phase as splenic tissue, azygos continuation (black arrow) of the inferior vena cava, and right renal vein draining into the azygos vein (arrowhead). Intrahepatic portion of the inferior vena cava is absent. Axial contrast enhanced CT image (c) shows, polysplenia at right upper quadrant (white arrows), absence of spleen at its normal location, liver located at midline related to situs ambiguous, and dilated azygos vein (black arrow).

Situs ambiguous with polysplenia, also referred to as left isomerism or bilateral left sidedness, is a complex congenital syndrome associated with multiple highly variable cardiovascular and visceral anomalies and is more common in females. Splenic number can vary from two to six and diameter from 1 cm to 6 cm. Location of the spleens may be either in the left or right side of the upper quadrant. Other abdominal anomalies coexisting with polysplenia include a right-sided stomach, a midline or left-sided liver, malrotation of the intestine, a short pancreas, absence of gallbladder, and inferior vena cava anomalies. The most common and characteristic inferior vena cava (IVC) anomaly is interruption of the infrahepatic portion of the IVC with azygos continuation (Figure 9). Radiologists should be aware of these rare syndromes and these coexisting anomalies to avoid misinterpreting them as separate pathological processes [1, 2, 6]. 

## 5. Splenosis

Splenosis is an acquired anomaly of the spleen which occurs in case of splenic trauma or splenectomy. Heterotopic autotransplantation and implantation of the splenic tissue can be located anywhere in the peritoneal cavity and even in extraperitoneal locations and other unusual locations including pleural cavity, lung, pericardium, pelvis, and subcutaneous tissues. Splenosis is often numerous and variable in size and shape. Thoracic splenosis requires a simultaneous splenic and left diaphragmatic injury that causes pleural or peripheral pulmonary nodule(s). Although both accessory spleen and splenosis are ectopic splenic tissues, their microscopic architecture, arteries supplying them, and origins are different. Accessory spleen which is a congenital anomaly has normal splenic tissue histology and is supplied by the branches of the splenic artery. However, splenosis is an acquired condition, has a distorted microscopic architecture, and is supplied by surrounding vessels. 

**Figure 10:**
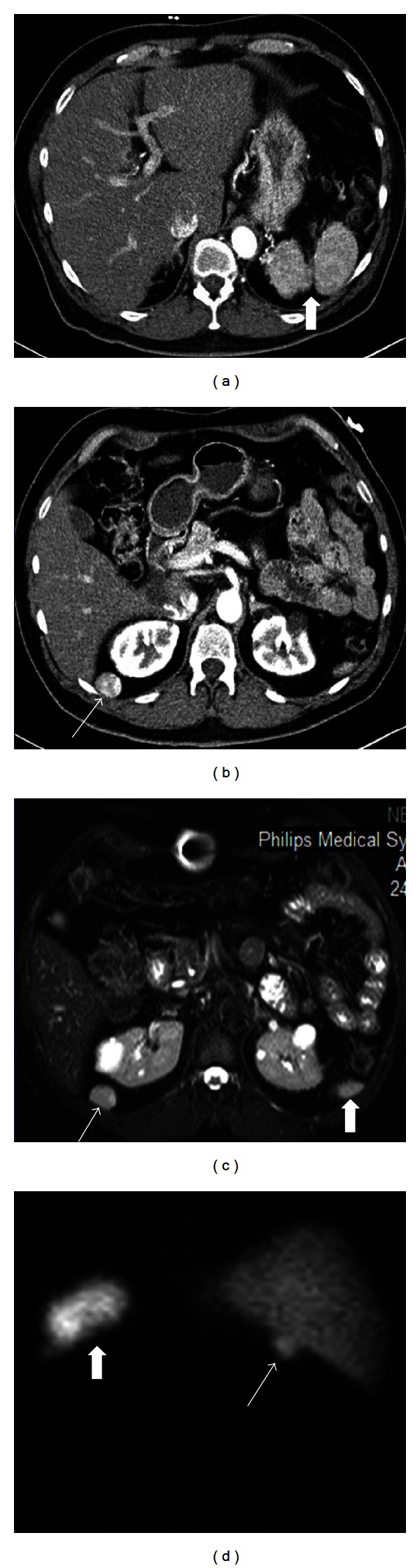
Splenosis in a 62-year-old male patient with a history of splenic trauma and splenectomy. Axial contrast enhanced CT images (a, b) show two splenic fragments (thick arrow) on the left upper quadrant (a) and a nodular mass (arrow) with a heterogenous contrast enhancement pattern as splenic tissue posterior to the liver and kidney (b). Axial T2 weighted image (c) demonstrates similar signal intensity of both the nodular mass (arrow) and inferior pole of the splenic fragments (thick arrow). Technetium-99 m posterior scintigraphy image (d) also shows uptake of the nodular mass (arrow) as splenic tissues on the left (thick arrow).

Splenosis is mostly asymptomatic and found incidentally on US, CT, and MRI examinations (Figure 10). With these imaging methods, it is often difficult to distinguish splenosis from other pathologic processes. Locations of splenosis especially along the hepatic surface or in the hepatic parenchyma, colonic surface or wall, and a pleural or lung nodule can mimic neoplastic lesions and may lead to an unnecessary surgery. It is important to know if the patient had a clinical history of splenic trauma or surgery that can be a clue to consider splenosis. There are also choices of methods to diagnose splenosis without invasive procedures including scintigraphy with Tc-99 m sulfur colloid, Tc-99 m heat-damaged erythrocytes and In-111 labeled platelets and MRI with intravenous administration of superparamagnetic iron oxide (SPIO). 

On the other hand, a functioning splenic tissue is a desired condition in case of splenectomy to maintain normal immunological function and eliminate the aged blood cells. But in a patient who had splenectomy for hypersplenism, a preservable splenic tissue is an undesired condition that may cause recurrent disease [11–13]. 

## 6. Small Spleen

**Figure 11:**
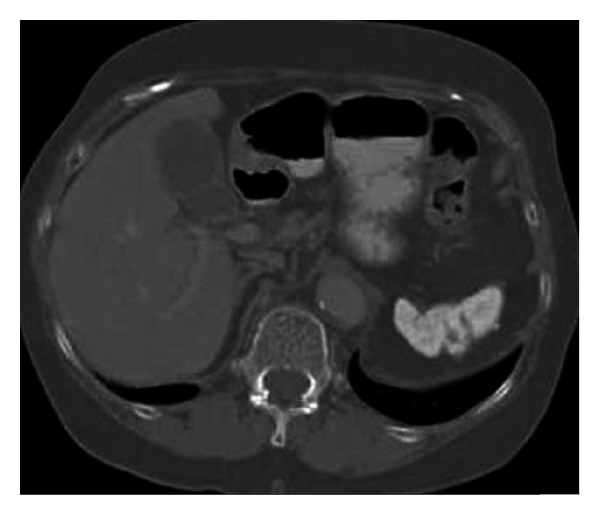
Axial contrast enhanced CT image at bone window demonstrates small and calcified spleen of a patient with sickle cell disease.

Adults with small spleen usually have homozygous sickle cell disease which causes chronic splenic infarction. The spleen can be densely calcified and small on radiographs and CT (Figure 11). Splenic size may be as small as 0.5–1 cm in case of autosplenectomy, and also can show decreased signal intensity on T1 and T2 weighted MR images related to iron deposition from chronic blood transfusions and/or calcifications [3, 4].

## 7. Diagnostic Pitfalls

**Figure 12:**
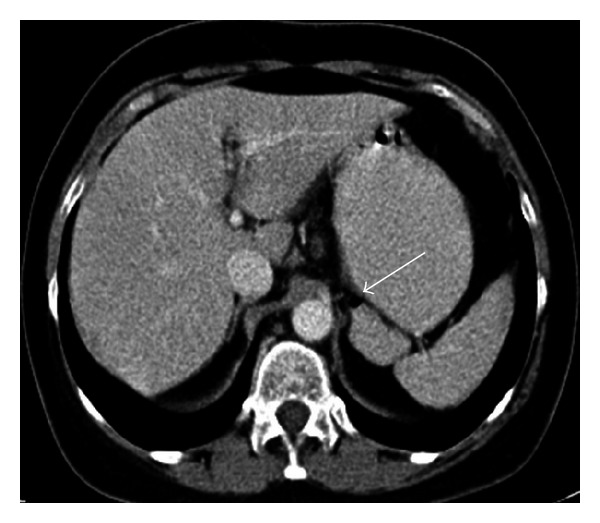
Axial contrast enhanced CT image with oral contrast medium reveals a hyperdense mass posterior to the stomach and medial to the spleen. The gas bubble anterior to the mass (arrow) helps gastric diverticulum in differentiating it from an accessory spleen.

There are also diagnostic pitfalls in assessing anatomical variations of spleen including splenic notches and clefts mimicking splenic laceration as mentioned before, an accessory liver lobe mimicking an accessory spleen or an intra-abdominal tumor, and a wandering accessory spleen mimicking an intra-abdominal mass. A gastric diverticulum located on the greater curvature may also mimic an accessory spleen when totally filled with oral contrast medium (Figure 12). 

**Figure 13:**
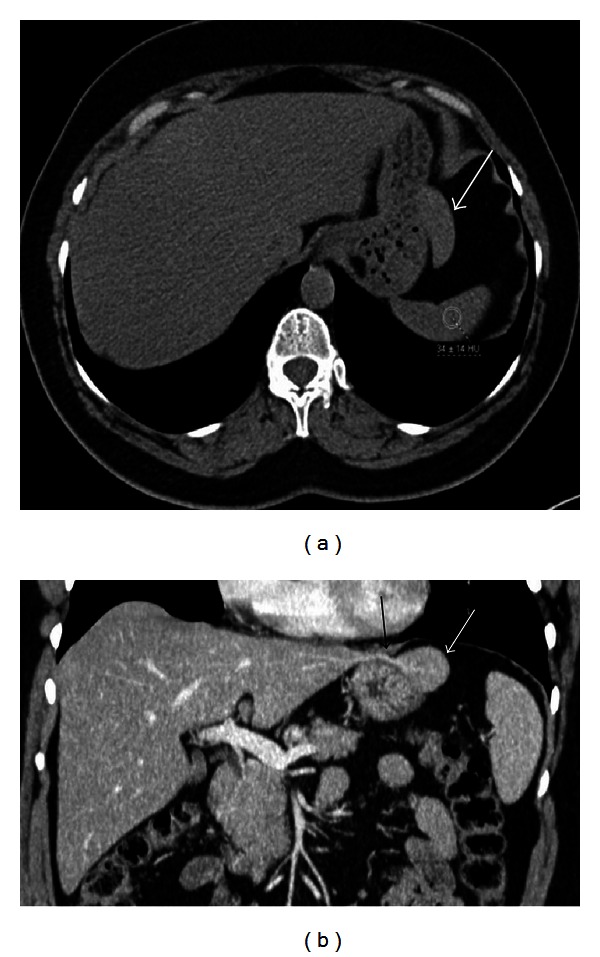
Nonenhanced axial CT image (a) of a 48-year-old female patient reveals a soft tissue mass (arrow) nearby greater curvature of the stomach. On coronal reformatted contrast enhanced CT image, there is a stalk (black arrow) between left lobe of the liver and the mass (white arrow) indicating accessory liver.

Accessory liver lobe is a very rare anatomic variation in which accessory liver tissue attaches to the normal liver tissue by a bridge of hepatic tissue, a mesentery, or a stalk (Figure 13). This rare entity is usually asymptomatic but occasionally could also present with acute abdomen in case of torsion. It can be diagnosed by using multidetector CT imaging providing multiplanar reconstruction, 3-dimensional, and maximum intensity projection images to show its connection with the liver [14–16]. 

**Figure 14:**
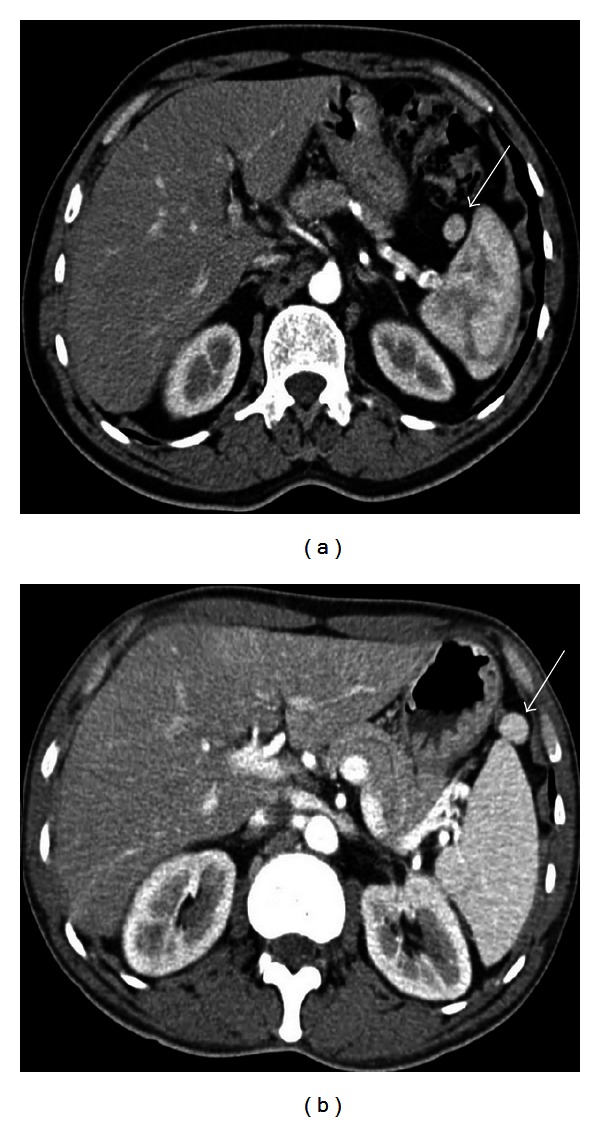
Axial contrast enhanced CT images (a), (b) of a 54-year-old male patient show wandering accessory spleen (arrow) anterior to the spleen which is at different locations in consecutive examinations.

A wandering accessory spleen is another extremely rare entity in which an accessory spleen has a long vascular pedicle with a normally located spleen (Figure 14). Although accessory spleen is a common entity in the population and mostly asymptomatic, a wandering accessory spleen poses risk for torsion and can be present with different degrees of abdominal pain related to its intermittent or acute torsion. And in case of a torsion depending on the location, a wandering accessory spleen can mimic a neoplastic mass, an abscess, or an organized hematoma. Imaging with US, CT, and MRI usually shows an inflammatory and avascular mass, and the exact diagnosis requires surgical exploration. This entity should also be included in the list of differential diagnosis of an intraperitoneal inflammatory mass despite its rarity [17–19]. 

In conclusion, knowing imaging findings of splenic variations and anomalies is important to avoid diagnostic pitfalls and misinterpretations.
